# Chikungunya Outbreak in Guangdong Province, China, 2010

**DOI:** 10.3201/eid1803.110034

**Published:** 2012-03

**Authors:** De Wu, Jie Wu, Qiaoli Zhang, Haojie Zhong, Changwen Ke, Xiaoling Deng, Dawei Guan, Hui Li, Yonghui Zhang, Huiqiong Zhou, Jianfeng He, Linghui Li, Xingfen Yang

**Affiliations:** Center for Disease Control and Prevention, Guangdong, China (D. Wu, J. Wu, D. Zhong, C. Ke, X. Deng, D. Guan, H. Li, Y. Zhang, H. Zhou, J. He, L. Li, X. Yang);; Dongguan Center for Disease Control and Prevention, Dongguan, China (Q. Zhang)

**Keywords:** emerging infectious diseases, chikungunya virus, viruses, chikungunya, infectious disease outbreak, ELISA, mosquitoes, immunoglobulin, dengue, arboviruses, reverse transcription PCR, China

## Abstract

A disease outbreak with dengue-like symptoms was reported in Guangdong Province, China, in October 2010. Testing results confirmed that the pathogen causing the outbreak was chikungunya virus. Phylogenic analysis indicated that this virus was a member of the Indian Ocean clade of the East/Center/South African subgroup of chikungunya virus.

Chikungunya virus (CHIKV) is a mosquito-borne virus that causes fever, headache, rash, nausea, vomiting, myalgia, and arthralgia, and has had a major effect on human health (*1*,*2*). The first human infections caused by CHIKV were reported ≈60 years ago (1952–1953) in eastern Africa (*3*). CHIKV has now become a worldwide public health problem. Although this virus is indigenous to tropical Africa, outbreaks of CHIKV fever have been reported in countries in the Indian Ocean region and Southeast Asia (*4*–*6*). With an increase in global travel, the risk for spreading CHIKV to regions in which the virus is not endemic has increased (*7*).

Multiple sporadic cases of nonindigenous CHIKV infection have been reported in China. In 1987, CHIKV was isolated from the serum of a patient, and antibodies against CHIKV were detected in a second, convalescent-phase patient in Yunnan Province (*8*). Four imported case of CHIKV infection confirmed by reverse transcription PCR (RT-PCR) were detected in Guangzhou and Moming, Guangdong Province, in travelers returning from Sri Lanka and Malaysia in 2008 (*9*,*10*). Another imported case from India was confirmed by using RT-PCR in our laboratory in 2009. We report an outbreak of CHIKV fever that occurred in Guangdong Province, China, in 2010.

## The Study

Guangdong Province is located in a subtropical zone. It has a high relative humidity, an average yearly temperature of 19°C–24°C, and an average yearly rainfall of 1,300–2,500 mm. *Aedes albopictus* mosquitoes are abundant and widespread. However, *Ae*. *aegypti* mosquitoes are found only in western Guangdong Province and not in the region around the city of Dongguan. In the months before the outbreak, the weather in Guangdong Province was particularly rainy.

During September 2010, patients reporting an illness with dengue-like symptoms were recorded by local community clinics in the suburbs of Dongguan, Guangdong Province. For epidemiologic investigation, the Guangdong Center for Disease Control and Prevention defined a clinical case of CHIK fever as a case characterized by sudden onset of fever with arthralgia, maculopapular rash, or myalgia. We identified 173 patients (74 male and 99 female patients) 2–93 years of age in 2 adjacent villages who had similar symptoms. More than 85% of the patients were found in these 2 villages in 97 families (>2 cases per family in 50 families).

**Figure 1:**
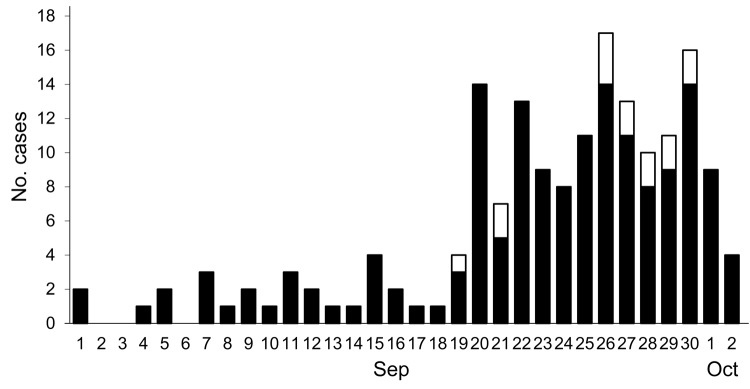
Cases of chikungunya infection in Guangdong, China, September 1–October 1, 2010. Black bar sections indicate clinical cases and white bar sections cases confirmed by molecular analysis.

The first patient became ill on September 1, and the number of CHIKV fever cases rapidly increased after September 19 (Figure 1), indicating an outbreak of CHIKV infections in the region. The outbreak spanned 2 months, and the peak occurred at the end of September/early October. None of the patients or any family members reported travel abroad since July 2010. No deaths were reported as a result of the outbreak, and most patients recovered within 1 week after onset of symptoms. No patients were hospitalized; however, several elderly patients reported joint pain after 2 weeks.

Densities of *Ae. albopictus* mosquitoes were investigated during the outbreak, and an especially high Breteau index of 77–180 was observed. The abundant rainfall likely resulted in an extremely high mosquito density. To control the outbreak, mosquito control measures were implemented and quarantine of patients with acute disease was enforced.

To identify the pathogen causing the outbreak, we collected 15 serum samples from 12 patients with acute disease and 3 patients with convalescent-phase disease who had dengue-like symptoms. Patient serum was assayed for CHIKV nucleic acid, antibody, and virus. DNA sequence analysis of amplified CHIKV envelope 1 (E1) was performed to infer possible source of transmission. Specimens were tested by real-time RT-PCR for CHIKV (*11*) and dengue virus.

Ten serum samples were positive for CHIKV. Virus-specific IgM and IgG were detected by IgM and IgG capture ELISAs (IBL, Hamburg, Germany). Seven samples were positive for IgM and 1 sample was positive for IgG (Table). There were 3 case-patients in whom CHIKV nucleic acid and antibody were found at the same time; 2 of these were in serum samples obtained 3–4 days after these samples were found to be positive for CHIKV IgM. We infer that high cross-reactivity in the ELISAs might contribute to these results.

**Table:** Characteristics of case-patients and serum sample detection for chikungunya virus, Guangdong, China, 2010*

Case-patient ID no.	Age, y/sex	Date of symptom onset, Sep 2010	Signs and symptoms							Test results		
			Fever	Red face	Headache	Arthralgia	Myalgia	MR		Virus isolation	Real-time RT-PCR/RT-PCR	IgM/ IgG
D10112	33/F	27	+	–	–	+	+	+		–	+/+	–/–
D10113	7/M	29	+	+	–	+	+	+		+	+/+	–/–
D10114	62/M	30	+	+	+	+	–	–		+	+/+	–/–
D10115	48/F	30	+	–	–	+	–	+		+	+/+	–/–
D10116	60/M	28	+	–	–	+	+	–		–	+/–	–/–
D10117	39/M	27	+	+	–	+	+	+		–	+/–	+/–
D10118†	59/M	19	+	+	–	+	–	+		ND	–/ND	+/+
D10119	59/F	26	–	+	–	+	–	+		ND	–/ND	–/–
D10120	10/F	26	+	–	+	–	–	+		ND	–/ND	+/–
D10121†	56/F	21	+	+	–	+	–	+		ND	–/ND	+/–
D10122†	24/F	21	+	+	–	+	+	+		ND	–/ND	+/–
D10123	3/F	26	+	–	–	–	–	+		–	+/–	–/–
D10124	60/M	26	+	–	–	+	+	+		–	+/+	–/–
D10125	60/F	29	+	–	–	+	+	+		–	+/+	+/–
D10126	39/M	28	+	–	–	+	+	+		–	+/+	+/–

*All samples were obtained on October 1, 2010. ID, identification; MR, maculopapular rash; RT-PCR, reverse transcription PCR; +, positive; –, negative; ND, not done. † Convalescent-phase case-patient.

**Figure A1:**
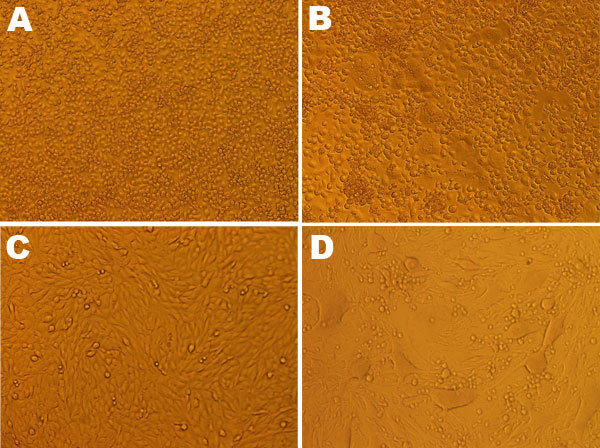
Effect of chikungunya virus nucleic acid–positive serum specimens on C6/36 and BHK-21 cells, Guangdong, China, 2010. A) Control (uninfected) C6/36 cells. B) Infected C6/36 cells, showing cytopathic effect. C) Control (uninfected) BHK-21 cells. D) Infected BHK-21 cells, showing cytopathic effect. (Original magnifications ×200.)

For phylogenetic analysis, RT-PCR was performed as described (*12*), and 7 amplicons were sequenced. The 10 nucleic acid–positive specimens were placed on C6/36 and BHK-21 cell lines to isolate CHIKV. Serum samples were 2-fold serially diluted 6 times (1:50–1:1,600) in minimal essential medium, and 1 mL of diluted sample was added to each well of a 24-well culture plate. Specimens were incubated at 33°C in an atmosphere of 5% CO_2_ and observed daily for <7 days for cytopathic effects (CPEs) (Figure A1). After specimens were incubated for 4–7 days, 3 CPEs were observed on C6/36 and BHK-21 cells. Development of CPEs in C6/36 cells is unusual for CHIKV. However, we observed the effect of C6/36 cell fusion on 3 specimens. We speculate that a virus mutation causes an increase in virulence or changes effects on infected C6/36 cells.

**Figure 2:**
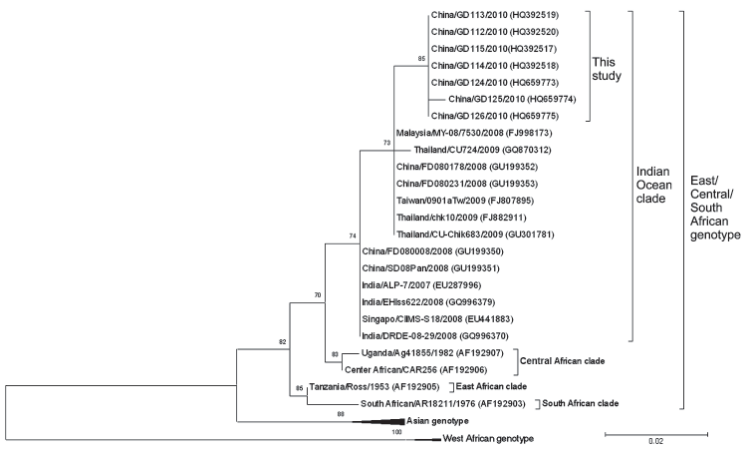
Phylogenetic analysis of partial envelope 1 gene sequences of chikungunya viruses, Guangdong, China, 2010. Numbers along branches indicate bootstrap values. GenBank accession numbers are indicated in parentheses. Scale bar indicates nucleotide substitutions per site.

Phylogenetic analysis was performed for partial E1 sequences (7 from this study and 24 from GenBank) by using MEGA5 (*13*). Nucleotide sequences were separated into 3 subgroups corresponding to the 3 globally circulating subgroups (Figure 2). Sequences of the 7 PCR products obtained in this study showed few differences from each other. Paired sequence identity ranged from 98% to 99% at the nucleotide level. Genetic analysis of the 325-nt fragment of E1 genes obtained in this study showed that all 7 sequences clustered in a unique branch within the Indian Ocean clade of the East/Central/South African (ECSA) genotype, and close to Thailand (GQ870312, FJ882911, GU301781), Malaysia (FJ998173), Taiwan (FJ807895), and China (GU199352, GU199353) isolates (98%–99%). The translated E1 gene fragment from 1 of the 7 isolates in this study (China/GD112/2010) had an expected 2-codon deletion. This deletion was also present in the ESCA clade but was not found in the other 6 isolates.

On the basis of sequence analysis, the highest degree of identity was observed with outbreak isolates and the E1 sequence from the Thailand strain (FJ882911) isolated in 2009. Paired identity values were 99% at the nucleotide level and 100% at the amino acid level. Nucleotide substitute analysis showed that a common nucleotide substitution was observed at partial E1 gene site 250 (T→C) in outbreak isolates and FJ882911. This substitution was not observed in other analyzed sequences from GenBank. These results suggested that the virus causing this outbreak was likely transmitted from a source in Southeast Asia and probably evolved from a strain that originated in Thailand.

## Conclusions

CHIKV was not endemic to China before 2010. However, in recent years, CHIKV strains from Southeast Asia with the ECSA genotype have been transmitted by infected persons to Guangdong Province. We report an outbreak of CHIKV fever in China. The low severity of the disease and misdiagnosis of dengue fever has likely encouraged widespread transmission of the virus. High-density mosquito populations and an immunologically uninfected population were 2 contributing factors in this outbreak.
